# An Integrative CGH, MSI and Candidate Genes Methylation Analysis of Colorectal Tumors

**DOI:** 10.1371/journal.pone.0082185

**Published:** 2014-01-27

**Authors:** Hassan Brim, Mones S. Abu-Asab, Mehdi Nouraie, Jose Salazar, Jim DeLeo, Hadi Razjouyan, Pooneh Mokarram, Alejandro A. Schaffer, Fakhraddin Naghibhossaini, Hassan Ashktorab

**Affiliations:** 1 Department of Pathology, Medicine and Cancer Center, Howard University, College of Medicine, Washington, DC, United States of America; 2 Histopathology Core, National Eye Institute, National Institutes of Health, Bethesda, Maryland, United States of America; 3 Section of Biomedical Computing, Clinical Center, National Institutes of Health, Bethesda, Maryland, United States of America; 4 Drexel University College of Medicine, Monmouth Medical Center, Department of Medicine, Long Branch, New Jersey, United States of America; 5 Biochemistry Department, Shiraz University of Medical Sciences, Shiraz, Iran; 6 National Center for Biotechnology Information, National Library of Medicine, National Institutes of Health, Bethesda, Maryland, United States of America; Vanderbilt University Medical Center, United States of America

## Abstract

**Background:**

Different DNA aberrations processes can cause colorectal cancer (CRC). Herein, we conducted a comprehensive molecular characterization of 27 CRCs from Iranian patients.

**Materials and Methods:**

Array CGH was performed. The MSI phenotype and the methylation status of 15 genes was established using MSP. The CGH data was compared to two established lists of 41 and 68 cancer genes, respectively, and to CGH data from African Americans. A maximum parsimony cladogram based on global aberrations was established.

**Results:**

The number of aberrations seem to depend on the MSI status. MSI-H tumors displayed the lowest number of aberrations. MSP revealed that most markers were methylated, except RNF182 gene. P16 and MLH1 genes were primarily methylated in MSI-H tumors. Seven markers with moderate to high frequency of methylation (SYNE1, MMP2, CD109, EVL, RET, LGR and PTPRD) had very low levels of chromosomal aberrations. All chromosomes were targeted by aberrations with deletions more frequent than amplifications. The most amplified markers were CD248, ERCC6, ERGIC3, GNAS, MMP2, NF1, P2RX7, SFRS6, SLC29A1 and TBX22. Most deletions were noted for ADAM29, CHL1, CSMD3, FBXW7, GALNS, MMP2, NF1, PRKD1, SMAD4 and TP53. Aberrations targeting chromosome X were primarily amplifications in male patients and deletions in female patients. A finding similar to what we reported for African American CRC patients.

**Conclusion:**

This first comprehensive analysis of CRC Iranian tumors reveals a high MSI rate. The MSI tumors displayed the lowest level of chromosomal aberrations but high frequency of methylation. The MSI-L were predominantly targeted with chromosomal instability in a way similar to the MSS tumors. The global chromosomal aberration profiles showed many similarities with other populations but also differences that might allow a better understanding of CRC's clinico-pathological specifics in this population.

## Background

Several studies have investigated the genetic and epigenetic events underlying the development of colorectal cancer (CRC). This is one of the world's leading common cancers [1]. Its incidence is different depending on the geographical and ethnic backgrounds. African-Americans, for example have the highest incidence of the disease in the US and have very high incidence when compared to their African counterparts with whom they share the same genetic background [2],[3],[4]. Most CRCs arise from adenomas, in a process described as adenoma-carcinoma sequence [5]. The initiation and progression of CRC is associated with alterations in the function of oncogenes and tumor suppressor genes.

Three major mechanisms affecting genes' functions in CRC have been described: microsatellite instability (MSI), chromosomal instability (CIN), and CpG island methylator phenotype (CIMP). More than one mechanism may occur in the same tumor. In MSI tumors, which account for 15% of CRCs, DNA mismatch repair genes are either mutated or methylated leading to tumors with a microsatellite instability phenotype [6].

In contrast, the CIN phenotype is characterized by global genomic aberrations resulting from deletions, amplifications and translocations of chromosomal fragments [7]. CIN results from specific mutations or regulatory silencing of gene silencing and could manifest as structural defects involving centromeres or centrosomes, microtubule dysfunction, telomere erosion, chromosome breakage and failure of cell cycle checkpoints [8].

In this study, we conducted a comprehensive study of Caucasian CRC tumors from Iranian patients, where CIN, MSI and DNA methylation have been considered. The mechanism of MSI was first characterized in the context of a subcategory of CRCs called hereditary non-polyposis colorectal cancer or Lynch syndrome, in which patients have heterozygous germline mutations of genes such as *MLH1* and *MSH2* [9]. The acquisition of recurrent chromosomal gains and losses during the progression from high-grade adenomas to invasive carcinomas has been repeatedly found in CIN CRC tumors [10] and other cancer genomes [11]. One of the earliest acquired genetic abnormalities during CRC progression involves chromosome 7 copy number gains which are observed in some colon adenomas as well [12]. At later stages of tumor progression, other specific chromosomal aberrations become more common, such as gains on chromosomes 8q, 20q [13], 7, 13 [14],[15] and copy number losses on chromosomes 8p, 17p, 18q [14],[16] 15q and 20q [17]. For some years, CIN and MSI tumors were considered as mutually exclusive, and it was thought that MSI tumors generally have stable, diploid karyotypes [18,[19]. However, recent studies have found that MSI and CIN can occur in the same tumor [20],[21]. Trautmann et al. found that at least 50% of MSI-H tumors have some degree of simultaneous chromosomal alterations [22]. Although evidence for some degree of CIN could be observed in the majority of MSI-H tumors, the pattern of specific gains and losses between MSI-H and MSS tumors is still poorly understood. MSI-H tumors tend to harbor gains of chromosomes 8, 12 and 13 and losses of 15q and 18q, while MSS tumors have a high degree and variable range of chromosomal aberrations [17],[22]. Chromosomal aberrations, like homozygous and heterozygous deletions or amplifications, alter the DNA copy number of large genomic regions or even whole chromosomal arms, leading to the inactivation of tumor suppressor genes or to the activation of oncogenes. Lassmann et al. studied 287 target sequences in Caucasian colorectal tumors and found aberrations in specific regions of chromosomes 7, 8, 13, 17, 20, [23]. Recently the list of these genes have been updated and carcinogenic pathways has been revealed [24].

Studies that explore differentially expressed genes that cause tumorigenesis or tumor development may lead to discovering specific targets for cancer therapy and increase our understanding of the process of tumorigenesis. We have previously showed [25],[26] that microduplications are mainly present in chromosomes 20q, 8q, and 7q while microdeletions occur in 18q, 8p and Xp in African American CRC tumors. We also showed evidence of possible linkage of MSI status with the nature of chromosomal aberrations along with a prevalent chromosome X amplification in male CRC patients [26].

Based on our previous studies [27],[28],[29],[30], we investigated the CIN, MSI and CIMP status in a cohort of Caucasian CRC patients and compared our results with the findings in German Caucasians [23] and in African Americans as well as with a list of colon cancer genes established by Sjöblom et al. based on their sequencing of 13,023 genes in 11 colon tumors [31]. We also performed a parsimony phylogenetic analysis of all recorded genomic aberrations to identify global genomic signatures that associate the oncogenic transformation. in the analyzed samples. The general aim of this study was to identify the chromosomal aberrations that point to specific genomic events in the analyzed population.

## Materials and Methods

### Ethics statement

The present study was approved by Shiraz University of Medical Sciences' Institutional Review Board. Written consent forms were obtained from all participants.

### Patients' selection

Fresh frozen archived samples were used. Colonic biopsies (n = 27) were obtained from CRC Caucasian patients undergoing surgery at Shiraz University Hospital, Shiraz, Iran. This study was approved by the local Institutional Review Board. Clinical data collected on each patient included gender, associated past medical history, medication use, and family history of colorectal cancer. Patients were deemed eligible if colonoscopy resulted in a first diagnosis of colon cancer, confirmed by histopathology. From the medical records, clinical information was collected and recorded based on the American Joint Committee on Cancer staging system.

### Samples' selection and Dna extraction

Fresh tumor blocks were cut into 5-µm thick sections on Superfrost slides (Fisher Scientific, Pittsburgh, PA). The tumor and normal areas were delineated by a pathologist using the matched hematoxylin and eosin stained slide. Microdissected slides were used for DNA extraction using Puregene kit according to the manufacturer's instructions (Qiagen, Germantown, MD). The goal of the microdissection was to minimize the cross-contamination of normal and tumor tissues, which could impact the outcome of the downstream experiments.

### MSI analysis

DNA from the analyzed tumors was used as a template in PCR reactions with five primer pairs, corresponding to the standard panel for MSI detection in colon cancer samples (BAT25, BAT26, NR21, NR22 and NR27), as described previously [32],[33],[34]. Samples that showed at least two PCR fragments with sizes different from the wild type were labeled microsatellite instability high (MSI-H), those with only one instability marker were labeled microsatellite instability low (MSI-L) while those with all PCR fragments with the expected size were labeled as microsatellite stable (MSS) [32],[33],[34].

### Methylation-specific PCR

The promoter methylation status of 15 cancer related genes (CAN genes) was determined as described previously [27]. The sequences of primers used for amplification of the promoter regions of each of the CAN genes were previously described [29] (See Table S1). Briefly, the PCR conditions were as follow: hotstart Taq polymerase (Qiagen) used with initial activation and denaturation 95°C for 15 min; 35 cycles [95°C for 45 sec; 60°C for 45 sec; 72°C for 1 min[ followed by a final extension 72°C for 10 min. In vitro methylated DNA and unmethylated lymphocytes DNA were used as positive and negative controls, respectively. The annealing temperature was 56°C for APC2 and CD109 [10] and 60°C for all other genes.

### Comparative genomic hybridization (CGH) experiments

In these experiments, we studied the chromosomal aberration profiles in the 27 CRC tumors. Our reference control was commercially procured sex-matched normal DNA (Promega, Wisconsin, WI). Tissues were evaluated by a GI pathologist for analysis of histological features including the size, type, location and pathological criteria of the carcinomas. An oligo microarray-based chip containing 105,000 human probes (Agilent, Santa Clara, CA; www.agilent.com) was used for CGH analysis. For each experiment, 1.5 µg of reference DNA and 1.5 µg of tumor DNA were used. Briefly, the test and reference DNAs were separately digested with *Alu* I and *Rsa* I (Promega, Madison, WI), and purified with the QIAprep Spin Miniprep kit (QIAGEN, Germantown, MD). Digested test and reference DNA were then labeled by random priming with Cy5-dUTP and Cy3-dUTP, respectively, using the Agilent Genomic DNA Labeling Kit Plus. Following the labeling reaction, the individually labeled test and reference samples were concentrated using Microcon YM-30 filters (Millipore, Billerica, MA) and then combined. Following probe denaturation and pre-annealing with *Cot-1* DNA, hybridization was performed at 65°C with rotation for 40 hours at 20 rpm. Four steps were done with Agilent Oligo CGH washes: wash buffer 1 at room temperature for 5 min, wash buffer 2 at 37°C for 1 min, an acetonitrile rinse at room temperature for 1 min and a 30 sec wash at room temperature in Agilent's Stabilization and Drying Solution. All slides were scanned on an Agilent DNA microarray scanner. Data including Copy Number Variations were obtained by Agilent Feature Extraction software 9 and analyzed with Agilent Genomic Workbench 5.0 software, using the statistical algorithms z score and ADM-2 according to sensitivity threshold respectively at 2.5 and 6.0 and a moving average window of 0.2 Mb. Mapping data were analyzed on the human genome sequence using the NCBI database build 35 also known as hg17 (http://www.ncbi.nlm.nih.gov).

### Computational analysis of genes targeted by copy number aberrations

To determine whether specific genes were gained or lost in each tumor sample, we compared the genomic locations of those genes with the gained and lost intervals in the “IntervalBasedReport” produced for each case by the array CGH software. To do this comparison, we developed UNIX scripts and programs in C and Perl. Part of the IntervalBasedReport is the magnitude of each gain or loss, which enabled us to filter the data by order of their magnitude and keep only those events that were above the threshold of 1.2-fold for gains and below the threshold 0.8-fold for losses. These are highly sensitive thresholds that we did set to detect any recorded aberrations.

### Parsimony phylogenetic analysis of CGH microarray data

Microarray data analysis generally looks at specific genes of known relevance to the pathology at hand. Here, we have taken all chromosomal aberrations into consideration to conduct a parsimony phylogenetic analysis. This type of analysis groups specimens according to their shared aberrations and prefers the phylogenetic tree that has the least number of steps to explain the data distribution among the specimens–the most parsimonious tree or cladogram in phylogenetic terminology. Therefore, as a priori to carrying out the parsimony phylogenetic analysis, each aberration had to be scored as shared (given the score of 1) or unshared (given the score of 0). Briefly, to find out the distribution of aberrations for each specimen in relation to the total aberrations of all specimens the following procedure was carried out: all aberrations of all the cancer specimens were summed up and the duplicates removed; each specimen's aberrations list was compared to the total list of aberrations and each aberration scored as present (1) or absent (0), this polarity assessment produced a new data matrix of CGH data. The new data matrix was processed for maximum parsimony with MIX algorithm (of the PHYLIP analytical package to produce the cladograms [26],[35].

### Statistical analysis

Numerical data was expressed as mean ± standard deviation. Student's t-test or one-way analysis of variance (ANOVA) were used for comparison of means. Categorical variables were compared using the chi-square test. P-values less than 0.05 were considered significant.

Age of patients was a continuous variable, while gender, location, differentiation, stage, MSI, and CAN genes methylation were categorical variables. The distribution of categorical variables were shown by frequency table, and for age by computing standard means. Associations between CIN with age, race, gender, differentiation, MSI, stage and tumor location were evaluated using a chi square test. Statistical analysis was performed using the SPSS 19.0 software package (IBM Corp., Somers, NY, USA).

## Results

### Characteristics of the analyzed samples

Twenty seven patients' tumors were characterized in this study for MSI, CIN and the methylation profiles for 15 known methylation targets. The clinical and demographical characteristics of these patients are summarized in Table 1. The age range for these patients was from 28 to 81 years, with 5 patients below the age of 50. Most tumors were moderately to well differentiated. There was only one poorly differentiated tumor. There were 16 male and 11 female patients in this group. The tumors were distributed in 3 different stages: stage 1 (n = 6), stage 2 (n = 13) and stage 3 (n = 8). There was a higher prevalence of left sided tumors with only 5 right sided tumors (Table 1).

**Table 1:** Characteristics of the patients involved in this study.

Patient	Sample	Age	Sex	Stage	Location	Diff.	MSI	Aberr.#
1	3T	51	F	3	L	Well	S	462
2	4T	55	F	1	R	Moderately	H	6
3	8T	46	M	3	L	Well	S	13
4	9T	63	M	3	L	Well	S	1
5	12T	40	M	1	L	Well	L	139
6	16T	58	F	2	L	Well	S	17
7	18T	45	M	2	L	Well	S	57
8	22T	60	M	2	L	Well	L	374
9	25T	72	M	2	L	Well	S	147
10	28T	60	M	2	L	Well	S	18
11	29T	65	M	2	L	Well	H	55
12	31T	47	F	1	R	Moderately	H	10
13	32T	70	M	3	R	Poorly	H	7
14	35T	81	M	1	L	Well	S	154
15	36T	78	F	3	L	Moderately	S	43
16	38T	81	M	1	R	Well	S	244
17	40T	54	M	3	L	Moderately	S	66
18	42T	68	F	1	L	Well	S	31
19	44T	28	F	2	L	Well	L	827
20	46T	57	F	2	R	Moderately	H	13
21	47T	63	F	2	L	Moderately	S	12
22	49T	40	F	2	L	Well	L	37
23	53T	70	M	3	L	Moderately	S	9
24	58T	70	M	3	L	Moderately	S	30
25	71T	67	M	2	L	Moderately	S	67
26	72T	60	M	2	L	Moderately	S	91
27	77T	55	F	2	L	Well	S	5

Abbreviations: Diff.: Differentiation, Aberr.#: Aberrations number, MSI: Microsatellite Instability: S: Stable, L: Low, H: High.

doi:10.1371/journal.pone.0082185.t001

### MSI analysis

Using the 5 recommended markers for MSI analysis, we established that 18 analyzed tumors were stable, 4 were MSI-L and 5 were MSI-H with at least 2 markers displaying instability. The computation of the available clinical and pathological parameters with the MSI status revealed no correlation with tumor stage or patient's gender. However, there was a clear association of the MSI status with tumor location with most of the MSI-H tumors (80%) being right sided while all MSI-L tumors were left sided. A similar observation was noted for age and MSI status with MSI-H tumors being older than MSI-L ones (Table 2).

**Table 2:** MSI analysis and association with patients' pathological and demographical characteristics.

	MSI			
	Stable (n = 18)	Low (n = 4)	High (n = 5)	P value
**Median age (25–75% interquartile)**	63.0 (54.7–70.5)	40 (31–55)	57 (51–67)	0.03
**Gender, no (%)**				0.51
• Male	12 (66.7)	2 (50)	3 (60)	
• Female	6 (33.3)	2 (50)	2 (40)	
**Location, no (%)**				<0.001
• Right	1 (5.6)	0 (0)	4 (80)	
• Left	17 (94.4)	4 (100)	1 (20)	
**Stage**				0.46
• One	3	1	2	
• Two	8	3	2	
• Three	7	0	1	
• Four	0	0	0	

doi:10.1371/journal.pone.0082185.t002

### Genes methylation analysis

Fifteen known methylation target genes were analyzed to establish their methylation status in the analyzed samples and whether their methylation correlate with other characteristics of the tumors and patients. Table 3 depicts the genes' methylation frequencies (based on the % of methylated samples) and possible correlations with other parameters. APC2, PTPRD, EVL, GPNMB, MMP2 and SYNE1 were methylated in most samples. CHD5, p16, STARD8, LGR6, RET, CD109 were less frequently methylated while CD109, ICAM5 and RNF182 displayed the lowest rates of methylation in the analyzed samples. The differential methylation based on other clinical and pathological criteria is reported in Table 3 where significant p values are highlighted. Detailed methylation data for each gene in all samples are provided in Table S2.

**Table 3:** Cancer related genes' methylation and association with clinical and pathological parameters.

Gene	MSI		p	Gender		p	Location		p	Differentiation			p	Age		p
	Yes	NO		F	M		R	L		P	M	W		<60	≥60	
P16	60	4.5	**0.002** *	18.2	12.5	0.68	60	4.5	**0.02** *	100	20	6.2	**0.03** *	20	8.3	0.39
hMLH1	80	4.5	**0.000** *	27.3	12.5	0.33	80	4.5	**0.000** *	100	30	6.2	**0.03** *	26.7	8.3	0.22
SYNE1	100	100		100	100		100	100		100	100	100		100	100	
RNF182	0	0		0	0		0	0		0	0	0		0	0	
MMP2	100	100		100	100		100	100		100	100	100		100	100	
ICAM5	20	4.5	0.23	9.1	6.2	0.78	20	4.5	0.23	0	10	6.2	0.90	13.3	0	0.18
CHD5	80	36.4	0.07	36.4	50.0	0.48	60	40.9	0.43	100	40	43.8	0.51	46.7	41.7	0.79
CD109	20	31.8	0.60	18.2	37.5	0.28	40	27.3	0.57	0	30	31.2	0.80	26.7	33.3	0.70
GPNMB	60	86.4	0.17	72.7	87.5	0.33	60	86.4	0.17	0	80	87.5	0.09	80	83.3	0.82
EVL	100	77.3	0.23	90.9	75.0	0.29	80	81.8	0.92	100	100	68.8	0.12	80	83.3	0.82
RET	20	40.9	0.38	9.1	56.2	**0.01** *	20	40.9	0.38	0	40	37.5	0.73	40	33.3	0.72
STARD8	60	59.1	0.97	81.8	43.8	**0.04** *	60	59.1	0.97	0	60	62.5	0.46	53.3	66.7	0.48
LGR6	60	27.3	0.16	27.3	37.5	0.58	80	22.7	0.01	100	40	25	0.25	33.3	33.3	1.00
PTPRD	80	81.8	0.92	81.8	81.2	0.97	80	81.8	0.92	0	80	87.5	0.09	86.7	75	0.43
APC2	100	86.4	0.38	81.8	93.8	0.33	100	86.4	0.38	100	100	81.2	0.31	86.7	91.7	0.68

* Statistically significant.

doi:10.1371/journal.pone.0082185.t003

### Genomic alterations in various chromosomes

All chromosomes were affected by the genomic instability with Chr 13 as the least targeted one with only 37 aberrations and Chr.1 and 2 as the most affected chromosomes with 207 and 194 total aberrations respectively. For most chromosomes, deletions were more prevalent than amplifications (Table 4). Chromosome X aberrations were primarily amplifications in male patients and deletions in female patients. Table 5 displays possible associations of aberration numbers with other parameters. Gender, stage and tumor location were not correlated with chromosomal aberrations number. However, the tumors' MSI status seems to correlate with the number of genomic alterations. Statistically significant p values were found whether we considered only two groups of patients (MSI-H and non MSI-H) or three groups of patients (MSS, MSI-L and MSI-H). The data shows that MSI-H tumors have the lowest number of aberrations while MSI-L tumors have an aberration profile more similar to MSS tumors, if not with more genomic instability (Table 5).

**Table 4:** Number of aberrations per chromosomes in the analyzed samples.

Chr.#	Total	Amplif.	Del.	Amplif. Males	Amplif. Females	Del. Males	Del. Females
1	207	84	123	18	66	84	39
2	194	77	117	13	64	67	50
3	131	46	85	12	34	42	43
4	107	20	87	8	12	44	43
5	95	43	52	5	38	31	21
6	110	35	75	6	29	35	40
7	130	76	54	18	58	43	11
8	124	45	79	21	24	46	33
9	124	50	74	17	33	59	15
10	104	57	47	10	47	30	17
11	174	62	112	15	47	72	40
12	104	50	54	13	37	33	21
13	37	21	16	10	11	10	6
14	102	41	61	11	30	40	21
15	96	47	49	14	33	33	16
16	145	59	86	21	38	71	15
17	171	72	99	22	50	70	29
18	62	6	56	2	4	27	29
19	157	66	91	14	52	86	5
20	92	59	33	21	38	26	7
21	55	9	46	3	6	32	14
22	105	34	71	8	26	57	14
X	102	40	62	30	10	8	54
Y	114	1	113	1	-	113	-

doi:10.1371/journal.pone.0082185.t004

**Table 5:** Number of aberrations and associations with clinical and demographical data using Pearson correlation. Numbers in this table are reported as median (25–75% interquantile).

	Median (25–75)	P value
Gender		0.2
• Male	61.5 (14–145)	
• Female	17 (10–43)	
Location		0.3
• Right	10 (6.5–128)	
• Left	49 (16–141)	
Stage		0.4
• 1	85 (9–176)	
• 2	55 (15–119)	
• 3	21.5 (7.5–60.2)	
• 4	0	
MSI		0.02
• Stable	37 (12.75–105)	
• Low	256 (62.5–713.7)	
• High	10 (6.5–34)	
MSI		0.006
• Non-MSI-H	50 (16–148)	
• MSI	10 (6.5–34)	

doi:10.1371/journal.pone.0082185.t005

### Comparison of the aCGH data with the other Caucasians CAN genes list

#### A: Lassmann et al. cancer genes list

**Figure 1:**
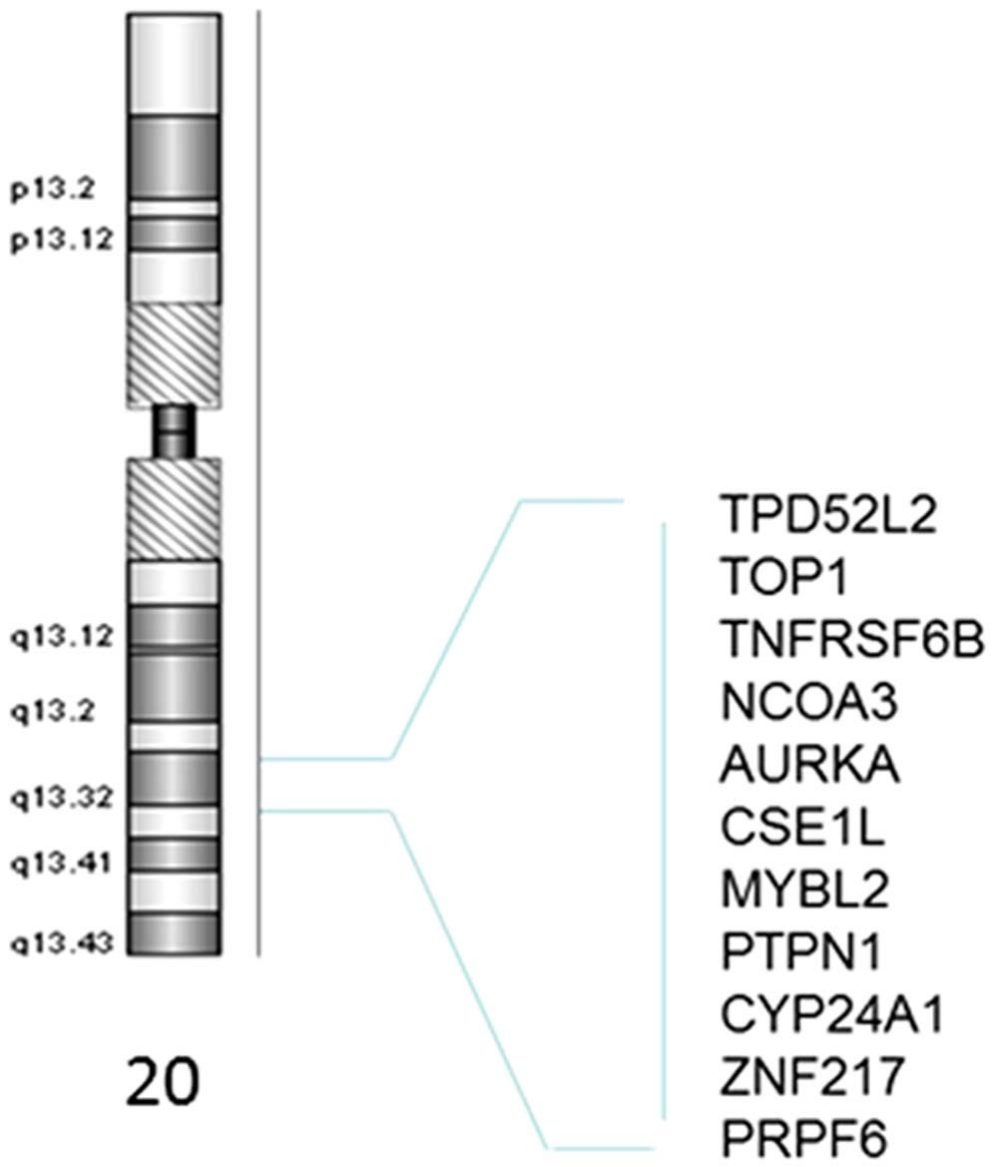
Chromosome 20's consistently amplified 20q13 region and its corresponding genes.

The CGH data for 41 oncogenes and tumor suppressor genes, were analyzed. The overall aberration profiles obtained with our samples were similar with a persistent amplification in chromosome 20 (Figure 1) that was obtained with our samples (Table S3). However, few differences for some genes are reported. RAF1, E2F5, EXT1, LRRC32, ATM, INS, DCC and KAL1 genes showed a different aberration profile in our samples when compared to Lassmann et al. samples profiles (Table S3). The most amplified markers were within 20qtel, 20q12 and 20q13 chromosomal locations (Figure 1). The most deleted markers were located on 17ptel, 17p12-17p11.2, 17p13.3, 18q21.3 and Xp22.3 chromosomal locations.

#### B: Sjoblom et al. cancer genes' list

The analysis of the aberration profiles of 68 genes identified by Sjoblom et al. [31] reveals that most of these markers are indeed subject to alterations in our study group (Table S4). The most amplified markers in this list are CD248, ERCC6, ERGIC3, GNAS, MMP2, NF1, P2RX7, SFRS6, SLC29A1 and TBX22. Most deletions were noted for ADAM29' CHL1, CSMD3, FBXW7, GALNS, MMP2, NF1, PRKD1, SMAD4 and TP53. Several markers, namely GALNS; GNAS;HIST1H1B; KRAS;MMP2;P2RY14;PHIP; RUNX1T1; SMAD2 and TGFBR2, showed different aberration profiles when compared to our previously published data with African American CRC patients (Table S4). Seven frequently methylated markers (Table 3) in this study (SYNE1, MMP2, LGR6, CD109, EVL, PTPRD and RET), that are also part of the Sjoblom et al. genes' list [31] showed low levels of chromosomal aberrations (Table S4).

### Phylogenetic analysis of the CRC aCGH data

**Figure 2:**
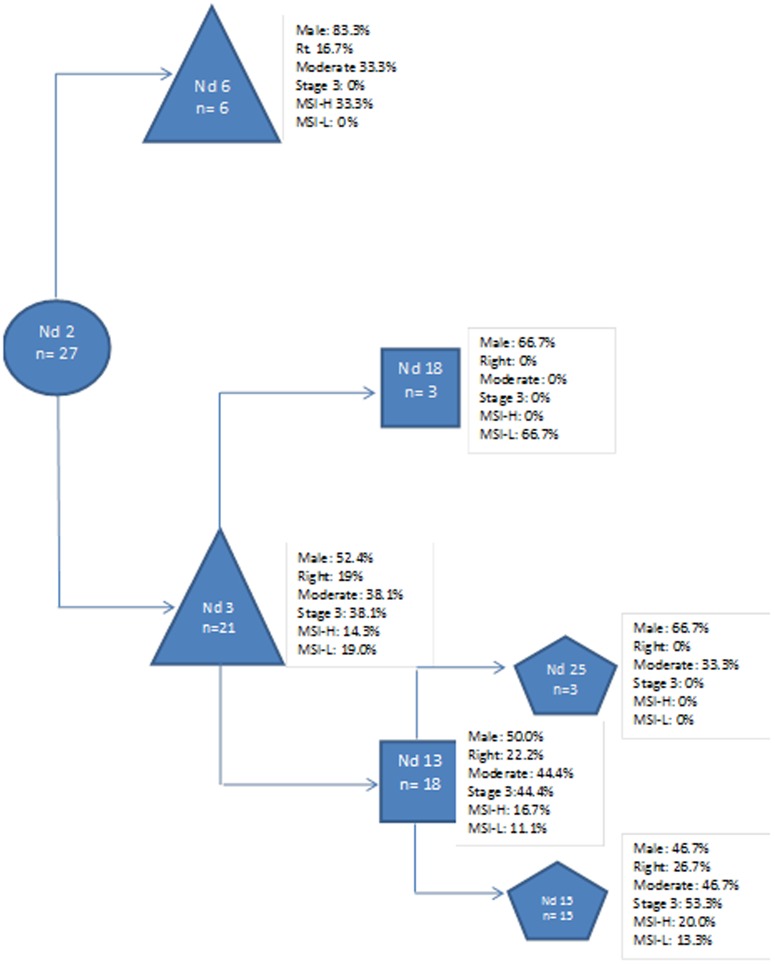
Cladogram depicting tumors' categorization based on Global chromosomal aberrations profiles: Nd#: refers to nodes number while n# refers to number of samples under the considered node, the characteristics of the samples under each node are depicted in the figure.

A parsimony analysis taking into account all recorded chromosomal aberrations led to a clustering of the different analyzed tumors (Figure 2). The generated clustering led to a distribution of the different tumors based on the nature of aberrations (Figure 2). The resulting clustering revealed a group of six samples defined by node 6. This node correspond to samples with aberrations predominantly in chromosomes 7 and X (Table S5). Five of the six cases under this node are males and present amplifications in chromosome X. Twenty one samples were represented under node 3. These cases presented a global chromosomal instability affecting most chromosomes (Table S5). It is noteworthy, that samples in node 15, a subnode of node 3, consist of 15 cases that include all stage 3 tumors in this study.

## Discussion

Genome-wide studies have the potential to reveal markers that may help explain clinical and pathological specifics of colorectal cancer in a given population. We have previously conducted several studies on the role of MSI, methylation of cancer related genes and mutations of known genes such as BRAF and KRAS, as well as microarray based studies of CRC tumors from different patients' populations [26],[27],[29],[30],[34],[36],[37]. These studies were instrumental in revealing some of the specific genetic and epigenetic alterations that lead to CRC. Herein, we elaborated upon our previous work and conducted a microsatellite instability analysis, methylation studies as well as whole genome analysis of copy number aberrations in CRC tumors from 27 Caucasian patients from Iran with the goal of understanding the interactions between these processes and compare the outcomes with other geographically remote CRC populations.

The analyzed samples came from a relatively young group of patients with 5 (out of 27 patients under the age of 50). While one would expect younger age to associate with MSI-H tumors because of their association to Lynch syndrome (an exclusion criteria for this study), our younger patients were more of the MSI-L phenotype. The association of younger age with MSI-L status was found to be statistically significant in this population (Table 2), pointing to a differential path leading to these tumors that is distinguishable from MSI-H tumors. All, but one, tumors were either well or moderately differentiated even though most were either stage 2 or 3 (Table 1). This points to a low metastatic potential of the CRC tumors in the analyzed population. Indeed, the methylation studies performed here reflect a low methylation frequency of ICAM5, a gene involved in cell-cell adhesion of which the methylation in other population was associated with aggressive, potentially metastatic tumors [29]. Most of the tumors (21 out of 27) were left sided pointing to a prevalence of distal colon biology in colon carcinogenesis in this population. The methylation analysis revealed that p16 and MLH1 methylation are primarily associated with right colon tumors (Table 3). Four of these right sided tumors were MSI-H, which indeed correlates with the methylation of these two markers that associate with the MSI-H status and the proximal tumor location. Aside from p16 and MLH1, 13 other genes were analyzed for methylation in this cohort and were all found to be methylated with the exception of RNF182. The methylation of RET gene was prevalent in male patients while that of STARD8 was more in female patients in a statistically significant manner. The methylation of the other genes did not show any association with demographic or pathological parameters. Seven of the methylation markers (SYNE1, MMP2, CD109, EVL, RET, LGR6 and PTPRD) with moderate to high methylation frequencies (Table 3) displayed low chromosomal aberrations (Table S4) pointing to the dominance of methylation as an altering process for these markers.

MSI-H tumors displayed the lowest number of chromosomal aberrations when compared to either MSS or MSI-L tumors (Table 4). Indeed, most MSI-H tumors result from a massive methylation process that targets DNA MMR genes, among others, leading to a higher rate of point mutations within the tumors rather than a global chromosomal instability. In contrast, MSI-L tumors have a very high number of chromosomal aberrations further dissociating them from the MSI-H tumors (Table 5). It is worth noting that MSI-L tumors were primarily distal while MSI-H were proximal. The difference in location as well as in the number of chromosomal aberrations clearly point to two different paths leading to these tumors [38]. Unlike our previously reported data in African American CRC patients [26], a parsimony analysis that took into consideration all reported aberrations did not lead to any specific clusters based on the MSI status. Both MSI-H (n = 5) and MSI-L (n = 4) tumors were scattered among the MSS tumor samples (Figure 2).

All chromosomes were subjects to alterations with chromosome 13 as the least and chromosome 1 as the most altered in all samples combined. There were also more deletions than amplifications. For most chromosomes, the number of deletions exceeded amplifications pointing to either a prevalence of more TSG than oncogenes in the genome or to the convenience for the deregulated carcinogenic cells to delete rather than carry over amplified chromosomal fragments. Only a chronological order of these alterations would allow a better understanding of the relevance of all of these alterations in the carcinogenic process. Many recent publications have reported the presence of driver and passenger genetic events in the carcinogenic process [24]. Such a classification is required to assess the relevance of any single genetic alteration in the path to cancer. Indeed, early events will have more decisive impact than those that accumulate down the road once the cell have lost its genetic integrity. A good example of this came through our parsimony analysis where all reported chromosomal aberrations were considered to cluster the different analyzed tumors. For six tumors that were predominantly from male patients (5 out of 6), the primary and major aberrations were on chromosomes 7 and X. This finding reflect the weight of chromosome 7 aberrations in early colon carcinogenesis. Also, most of the aberrations targeting chromosome X were amplifications and only in the 5 male patients in this group, consistent with our previously reported findings in male African American colon cancer patients [25],[26]. The parsimony analysis also revealed that most analyzed tumors (21 out 27) had a global chromosomal instability affecting most chromosomes. The eight patients with stage 3 clustered together and displayed highly altered karyotypes in our study (Figure 2).

Most amplified chromosomes were chr. 1, 2, 7, 16, 17, 19 and 20. This shows a different pattern than that reported in African Americans where chr. 3, 5, 7 and 8 were the primary targets for amplification. As for deletions, the primary chromosomal targets were 1, 2, 3, 4, 6, 8, 11, 16, 17, 19, 22 and X. Chromosome X deletions were primarily reported in female patients while the amplification for this chromosome were primarily in male patients further confirming our data with African Americans [26]. However, the deletions profile in the analyzed population seems also to differ from that in African Americans pointing to different CRC patients' profile in regards to chromosomal instability.

The aberration profile of 41 oncogenes and tumor suppressor genes in the analyzed population was compared to those obtained in German Caucasians and African American CRC patients (Table S3). Overall, the profiles were similar with a striking amplification of several markers on chr.20q arm in all three populations. Aberrations in this chromosome have already been linked to several other cancers including prostate, gastric and breast cancer [39],[40],[41]. Chromosome 20 amplification was specifically linked to invasive poor outcome breast cancers pointing to the presence of many oncogenes on this chromosome [42]. However, in the analyzed population, few markers showed a differential aberration profile. Indeed, RAF1 gene was primarily amplified in contrast to the German Caucasians. This marker was shown to be deleted in small cell lung cancers [43]. However, amplification, as in our population is more in line with its proto-oncogenic character [44]. The E2F5 gene was amplified in the two other populations while it was deleted in 18.5% of the analyzed samples (Table S3). The amplification is more in line with its identity as an oncogene in colon as well as in other cancers [45]. Some analyzed samples (22.2%) showed substantial deletion of the INS gene in contrast with the other two populations. However, amplification was also noted in 14.5% of cases. A similar profile of aberration was noted for KAL1 that was amplified in the other populations and targeted by both deletion and amplification in the analyzed population. Most publications report missense, antisense or deletions of this gene in the context of Kallmann syndrome, but none in the context of cancer [46]. As for the 18q arm DCC gene, it was primarily deleted as we reported for African Americans as well, in contrast with the German Caucasian patients. The deletion pattern fits the role of this marker as a TSG [47],[48].

Sjoblom et al. have sequenced breast and colorectal cancers [31] and established a list of 68 highly mutated genes of which we analyzed the aberration status in our samples. Recently the list of these genes has been updated and carcinogenic pathways have been revealed [24]. All of the genes showed some level of aberrations in the analyzed tumors, although at different levels and frequencies (Table S4). This finding further consolidates the status of these genes as primary targets of mutation in colorectal cancers. Many of these genes were also targeted by methylation. Of these genes, seven were analyzed for methylation (Table 3). These seven genes (SYNE1, PTPRD, LGR6, RET, EVL, CD109 and MMP2) showed moderate to high methylation frequencies in the analyzed samples but little chromosomal aberrations, if any, when compared to other markers known not to be preferential methylation targets.

GNAS and ERGIC3 were the most amplified markers. Both are located on the 20q arm. As for GNAS, this gene's regulation involves activating mutations, histone modifications [49] along with gene amplification. We recently found that GNAS promoter is also subject to hypomethylation [50] which reflect the collaboration of all known regulation processes towards more expression of this gene in the path to CRC. For ERGIC3 gene, its overexpression was associated with epithelial to mesenchymal transition in hepatocellular carcinoma cells where activating miR-490-3p targets this gene [51]. Other amplified markers were SFRS6, LMO7 and TBX22 on chromosomes 20, X and 13 q arm, respectively. These markers were also amplified in African American CRC tumors [26]. As pointed earlier, deletions were more prevalent genome wide and in this list of genes as well. At least 10 markers showed high levels of deletions (Table S4), among which TP53, SMAD4, GALNS, CSMD3 and CHL1 are the most prominent. The deletion of SMAD4 is consistent with its TSG status along other SMAD proteins [52]. Indeed, SMAD2 was also preferentially deleted in the analyzed samples. TP53, a known TSG, that has been extensively studied is often deleted in several cancers [53],[54]. GALNS gene mutations have been associated with Mucopolysaccharidosis [55]. However, its implication in colon cancer might occur at the level of mucin production by the tumors. It is noteworthy that the patients in the present study, while in advanced stages, none was metastatic which might have been facilitated by the GALNS deletion. Indeed, in comparison with African Americans, where we find many mucin producing potentially metastatic tumors, 46% displayed GALNS gene amplification. CSMD3 has been identified as a candidate gene for benign adult familial myoclonic epilepsy [56], however no correlation to colon cancer is available. As for CHL1 gene, its negative regulation by miR-10a promoted cell growth, invasion and migration in cervical cancer cells [57]. This reflects its TSG status and confirms the relevance of its deletion in 37% of the analyzed cases in stark contrast with African American CRC tumors (Table S4).

In conclusion, in this first comprehensive analysis of CRC tumors from Iranian patients, we report a high MSI rate of ∼18%. These MSI tumors displayed the lowest level of chromosomal aberrations but high methylation frequencies. The MSI-L, (∼17%) were predominantly targeted with chromosomal instability in a way similar to the MSS tumors. The global chromosomal aberration profiles showed many similarities with other populations but also differences that might allow a better understanding of the clinical and pathological specifics of CRC in this population.

## Supporting Information

Table S1 **MSP Primers for the CAN genes from Slobjom et al. study.**
(DOCX)

Table S2 **Detailed MSP data per genes and samples.**
(XLSX)

Table S3 **Comparison of this study's CGH data with those from Lassmann et al. genes' list.**
(DOC)

Table S4 **Comparison of this study's CGH data with CAN genes' Sjoblom list.**
(DOC)

Table S5 **Chromosomal aberrations defining** **Figure 2** **cladogram's nodes.**
(XLSX)
