# Supplementary material for: An Integrative CGH, MSI and Candidate Genes Methylation Analysis of Colorectal Tumors
Source: PLoS One. 2014 Jan 27;9(1):e82185. doi: 10.1371/journal.pone.0082185 (PMC3903472; doi:10.1371/journal.pone.0082185)

Supp. Table 1:used for Methylation Specific PCR for the 13 CAN genes from Slobjom et al.Study

(MLH1 and p16 primers have been used widely and are not in the table below).


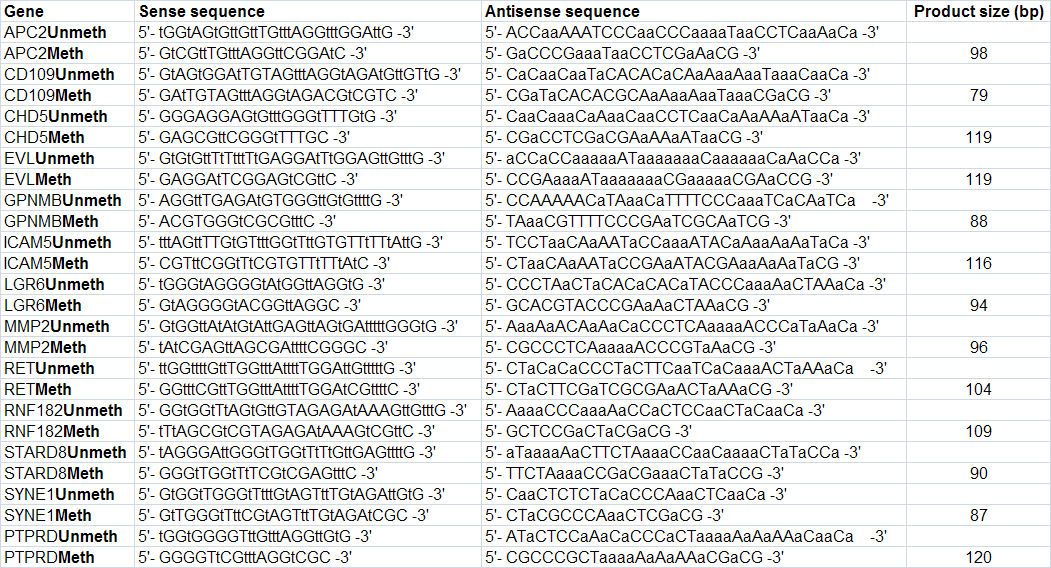

Supplement: Table S1 — (DOCX) [file pone.0082185.s001.docx]
