# Supplementary material for: An Integrative CGH, MSI and Candidate Genes Methylation Analysis of Colorectal Tumors
Source: PLoS One. 2014 Jan 27;9(1):e82185. doi: 10.1371/journal.pone.0082185 (PMC3903472; doi:10.1371/journal.pone.0082185)
Supplement: Table S3 — (DOC) [file pone.0082185.s003.doc]

Supp. Table 3: Comparison of this study’s CGH data with those from Lassmann et al. genes’ list.

|  |  | Lassmann et al. | | This study | |
| --- | --- | --- | --- | --- | --- |
| Gene | Location | Amplified (%) | Deleted (%) | Amplified (%) | Deleted (%) |
| THRB | 3p24.3 |  | 32 | 0 | 14.8 |
| RAF1 | 3p25 |  | 14 | 22.2 | 3.7 |
| RFC2 | 7q11.2 | 36 |  | 22.2 | 0 |
| CYLN2 | 7q11.23 | 36 |  |  |  |
| MET | 7q31 | 23 |  | 22.2 | 3.7 |
| LPL | 8p22 |  | 23 | 0 | 18.5 |
| E2F5 | 8q22-q21.3 | 36 |  | 3.7 | 18.5 |
| EXT1 | 8q24.11-q24.13 | 32 |  | 3.7 | 11.1 |
| MYC | 8q24.12-q24.13 | 36 |  | 7.4 | 0 |
| EGR2 | 10q21.3 |  | 23 | 0 | 14.8 |
| DMBT1 | 10q25.3-q26.1 |  | 23 | 3.7 | 7.4 |
| LRRC32 | 11q13.5 | 32 |  | 3.7 | 0 |
| ATM | 11q22.3 | 27 |  | 0 | 14.8 |
| INS | 11p tel | 32 |  | 14.8 | 22.2 |
| BRCA2 | 13q12-q13 | 36 |  | 25.9 | 3.7 |
| RB1 | 13q14 | 41 |  | 25.9 | 3.7 |
| MAP2K5 | 15q23 |  | 32 | 3.7 | 11.1 |
| SP6 | 17ptel |  | 23 | 14.8 | 33.3 |
| TOP3A | 17p11.2 |  |  | 7.4 | 29.6 |
| LLGL1 | 17p12-17p11.2 |  | 36 | 7.4 | 29.6 |
| FLII | 17p12-17p11.2 |  | 23 | 7.4 | 29.6 |
| HIC1 | 17p13.3 |  | 32 | 11.1 | 29.6 |
| CTDP1 | 18q tel |  | 45 | 3.7 | 33.3 |
| LAMA3 | 18q11.2 |  | 14 | 0 | 44.4 |
| BCL2 | 18q21.3 |  | 23 | 0 | 40.7 |
| DCC | 18q21.3 | 32 | 18 | 0 | 44.4 |
| TPD52L2 | 20qtel | 27 |  | 59.2 | 0 |
| TOP1 | 20q12-q13.1 | 32 |  | 51.8 | 0 |
| TNFRSF6B | 20q13 | 32 |  | 51.8 | 0 |
| NCOA3 | 20q13 | 32 |  | 51.8 | 0 |
| AURKA | 20q13 | 36 |  | 51.8 | 0 |
| CSE1L | 20q13 | 27 |  | 51.8 | 0 |
| MYBL2 | 20q13.1 | 32 |  | 55.5 | 0 |
| PTPN1 | 20q13.1-q13.2 | 23 |  | 59.2 | 0 |
| CYP24A1 | 20q13.2 | 36 |  | 51.8 | 0 |
| ZNF217 | 20q13.2 | 32 |  | 55.5 | 0 |
| PRPF6 | 20q13.3 | 27 |  | 44.4 | 0 |
| PCNT | 21qtel |  | 18 | 7.4 | 11.1 |
| XIST | Xq13.2 | 36 |  | 25.9 | 22.2 |
| STS | Xp22.3 |  | 23 | 25.9 | 22.2 |
| KAL1 | Xp22.3 | 36 |  | 29.6 | 25.9 |
