# Supplementary material for: An Integrative CGH, MSI and Candidate Genes Methylation Analysis of Colorectal Tumors
Source: PLoS One. 2014 Jan 27;9(1):e82185. doi: 10.1371/journal.pone.0082185 (PMC3903472; doi:10.1371/journal.pone.0082185)
Supplement: Table S4 — (DOC) [file pone.0082185.s004.doc]

Supp. Table 4: Comparison of this study’s CGH data with CAN genes’ Sjoblom list

|  |  | This study | |
| --- | --- | --- | --- |
| Gene | Location | Amplified (%) | Deleted (%) |
| ABCA1 | 9q31.1 | 1 (3.7) | 1 (3.7) |
| ACSL5 | 10q25 | 0 | 2 (7.4) |
| ADAM29 | 4q34 | 0 | 6 (22.2) |
| ADAMTS15 | 11q25 | 1(3.7) | 1 (3.7) |
| ADAMTS18 | 16q23 | 4 (14.8) | 0 |
| ADAMTSL3 | 15q25.2 | 1(3.7) | 3 (11.1) |
| APC | 5q22 | 0 | 4 (14.8) |
| C10orf137 | 10q26.1 | 2 (7.4) | 2 (7.4) |
| C15orf2 | 15q11 | 3 (11.1) | 3 (11.1) |
| **CD109** | 6q13 | 0 | 3 (11.1) |
| CD248 | 11q13 | 5(18.5) | 1 (3.7) |
| CD46(MCP) | 1q32 |  |  |
| CHL1 | 3p26.1 | 1(3.7) | 10 (37.0) |
| CNTN4 | 3p26 | 0 | 4 (14.8) |
| CSMD3 | 8q23.3 | 1(3.7) | 6 (22.2) |
| EPHA3 | 3p11.2 | 0 | 5 (18.5) |
| EPHB6 | 7q34 | 4 (14.8) | 0 |
| ERCC6 | 10q11.2 | 2 (7.4) | 1 (3.7) |
| ERGIC3(SBDCAG84) | 20q12 | 15 (55.5) | 0 |
| **EVL** | 14q32.2 | 3 (11.1) | 3 (11.1) |
| EXOC4(SEC8L1) | 7q31 | 4 (14.8) | 0 |
| EYA4 | 6q23 | 0 | 3 (11.1) |
| FBXW7 | 4q31.3 | 1(3.7) | 6 (22.2) |
| GALNS | 16q24.3 | 3 (11.1) | 7 (25.9) |
| GNAS | 20q13.3 | 15 (55.5) | 0 |
| GUCY1A2 | 11q22 | 0 | 3 (11.1) |
| HAPLN1 | 5q14.3 | 0 | 4 (14.8) |
| HIST1H1B | 6p22 | 2 (7.4) | 0 |
| KCNQ5 | 6q14 | 0 | 3 (11.1) |
| KIAA1409 | 14q32.1 | 2 (7.4) | 5 (18.5) |
| KRAS | 12p12.1 | 2 (7.4) | 2 (7.4) |
| KRT73(K6IRS3) | 12q13.3 | 4 (14.8) | 0 |
| **LGR6** | 1q32.1 | 4 (14.8) | 0 |
| LMO7 | 13q22.2 | 7 (25.9) | 2 (7.4) |
| LRP2 | 2q31 | 0 | 3 (11.1) |
| MAP2 | 2q34-35 | 0 | 4 (14.8) |
| ACTL9 | 19p13.2 | 4 (14.8) | 3 (11.1) |
| MKRN3 | 15q11 | 0 | 3 (11.1) |
| MLL3 | 7q36.1 | 4 (14.8) | 1 (3.7) |
| **MMP2** | 16q12-13 | 5(18.5) | 6 (22.2) |
| NF1 | 17q11.2 | 8 (29.6) | 8 (29.6) |
| OBSCN | 1q42.1 | 3 (11.1) | 4 (14.8) |
| P2RX7 | 12q24 | 5(18.5) | 0 |
| P2RY14 | 3q25 | 0 | 3 (11.1) |
| PHIP | 6q14 | 0 | 4 (14.8) |
| PKHD1 | 6p12.2 | 1(3.7) | 3 (11.1) |
| PKNOX1 | 21q22.3 | 2 (7.4) | 3 (11.1) |
| PRKD1 | 14q11 | 0 | 6 (22.2) |
| **PTPRD** | 9p23-24 | 2 (7.4) | 5 (18.5) |
| PTPRU | 1p35 | 2 (7.4) | 5 (18.5) |
| **RET** | 10q11.2 | 3 (11.1) | 1 (3.7) |
| RUNX1T1 | 8q22 | 1(3.7) | 3 (11.1) |
| SCN3B | 11q23.3 | 1(3.7) | 1 (3.7) |
| SFRS6 | 20q13.1 | 15 (55.5) | 0 |
| SLC29A1 | 6p21 | 5(18.5) | 0 |
| SLC44A4(C6orf29) | 6p21.3 | 4 (14.8) | 2 (7.4) |
| SMAD2 | 18q21.1 | 0 | 10 (4.7) |
| SMAD3 | 15q22.3 | 3 (11.1) | 3 (11.1) |
| SMAD4 | 18q21.1 | 0 | 12 (44.4) |
| **SYNE1*** | 6q25 | 0 | 1 (3.7) |
| TBX22 | Xq21.1 | 8 (29.6) | 5 (18.5) |
| TCF7L2 | 10q25.3 | 0 | 2 (7.4) |
| TGFBR2 | 3p22 | 0 | 4 (14.8) |
| TP53 | 17p13.1 | 5(18.5) | 8 (29.6) |
| TTLL3 | 3p25.3 | 3 (11.1) | 1 (3.7) |
| UHRF2 | 9p24.1 | 2 (7.4) | 2 (7.4) |
| UQCRC2 | 16p12 | 4 (14.8) | 1 (3.7) |
| ZNF442 | 19p13.2 | 5 (18.5) | 3 (11.1) |

* Genes in bold have also been analyzed for promoter methylation.

Figures’ legends:

Figure 1: Chromosome 20’s consistently amplified 20q13 region and its corresponding genes

**Figure 2:** Cladogram depicting tumors’ categorization based on Global chromosomal aberrations profiles: Nd#: refers to nodes number while n# refers to number of samples under the considered node, the characteristics of the samples under each node are depicted in the figure. .

Supplemental Material:

Supplemental Table 1: MSP Primers for the CAN genes from Slobjom et al. study

Supplemental Table 2; Detailed MSP data per genes and samples.

Supplemental Table 3: Chromosomal aberrations defining figure 2 cladogram’s nodes.
